# Weight Loss After Sleeve Gastrectomy According to Metabolic Dysfunction-Associated Steatotic Liver Disease Stage in Patients with Obesity: A Liver Biopsy-Based Prospective Study

**DOI:** 10.3390/nu16223857

**Published:** 2024-11-12

**Authors:** José Ignacio Martínez-Montoro, Isabel Arranz-Salas, Carolina Gutiérrez-Repiso, Ana Sánchez-García, Luis Ocaña-Wilhelmi, José M. Pinazo-Bandera, Diego Fernández-García, Araceli Muñoz-Garach, Dieter Morales-García, Miren García-Cortés, Eduardo García-Fuentes, Francisco J. Tinahones, Lourdes Garrido-Sánchez

**Affiliations:** 1 Department of Endocrinology and Nutrition, Virgen de la Victoria University Hospital, 29010 Málaga, Spain; 2 Instituto de Investigación Biomédica de Málaga (IBIMA-Plataforma BIONAND), 29010 Málaga, Spain; 3 Centro de Investigación Biomédica en Red de Fisiopatología de la Obesidad y Nutrición (CIBEROBN), Instituto de Salud Carlos III, 28029 Madrid, Spain; 4 Department of Anatomical Pathology, Virgen de la Victoria University Hospital, 29010 Málaga, Spain; 5 Department of Human Physiology, Human Histology, Anatomical Pathology and Physical Education, University of Málaga, 29010 Málaga, Spain; 6 Department of General and Digestive Surgery, Virgen de la Victoria University Hospital, 29010 Málaga, Spain; 7 Department of Gastroenterology, Virgen de la Victoria University Hospital, 29010 Málaga, Spain; 8 Centro de Investigación Biomédica en Red de Enfermedades Hepáticas y Digestivas (CIBERehd), Instituto Salud Carlos III, 28029 Madrid, Spain; 9 Department of Endocrinology and Nutrition, Virgen de las Nieves University Hospital, 18014 Granada, Spain; 10 Department of Medicine and Dermatology, Faculty of Medicine, University of Málaga, 29010 Málaga, Spain

**Keywords:** metabolic dysfunction-associated steatotic liver disease (MASLD), metabolic dysfunction-associated steatohepatitis (MASH), liver biopsy, obesity, sleeve gastrectomy, weight loss

## Abstract

Background: The role of metabolic dysfunction-associated steatotic liver disease (MASLD) in sleeve gastrectomy (SG)-related outcomes remains uncertain. In this study, we aimed to assess the influence of preoperative biopsy-proven MASLD and its stages on weight loss after SG. Methods: One hundred sixty-three patients with obesity undergoing SG with concomitant intraoperative liver biopsy were followed up for 1 year. Fifty-eight participants were categorized as no MASLD, thirty-eight as metabolic dysfunction-associated steatotic liver (MASL), and sixty-seven as metabolic dysfunction-associated steatohepatitis (MASH). Percentage total weight loss (%TWL) and percentage excess weight loss (%EWL) 1 year after SG were calculated for the different groups. We also evaluated the association between preoperative MASLD (and its stages) and weight loss, after adjusting for potential confounders. Results: Significant differences among groups were detected in %EWL (*p* = 0.004, ANOVA test), but not in %TWL (*p* = 0.079). However, significant differences in %TWL were found when MASH and no MASH (i.e., participants with MASL and participants without MASLD) groups were compared (27.3 ± 9.9 vs. 30.7 ± 9, respectively, *p* = 0.025). In the linear regression model for predicting %EWL 1 year after SG, the presence of MASH was independently associated with a lower %EWL, after adjusting for age, sex, baseline body mass index (BMI), and baseline glycated hemoglobin (HbA1c) (Beta −7.1; 95% CI −13.6, −0.5; *p* = 0.035). The presence of MASLD, liver fibrosis, or advanced liver fibrosis (≥F2) was also associated with lower %EWL after SG in crude models, although they did not remain significant after adjusting for these confounders. The presence of MASH was inversely related to %TWL, although the association did not remain significant after adjustment (Beta −2.7; 95% CI −5.7, 0.2; *p* = 0.069). Conclusions: MASH may be independently associated with lower %EWL 1 year after SG in patients with obesity.

## 1. Introduction

Obesity is a chronic disease associated with major health, social, and economic burdens [1]. In the last decades, the prevalence of obesity has reached pandemic proportions, with over 800 million adults suffering from this disease worldwide [2], and it is expected that this rising trend will continue in the coming years [2,3].

Lifestyle interventions, including diet and physical activity, are the mainstay of treatment of obesity [4]. Additionally, some medications, such as glucagon-like peptide-1 (GLP-1) receptor agonists, can help to achieve and maintain weight loss [4]. However, bariatric surgery (BS), recommended for patients with a body mass index (BMI) ≥ 35 kg/m^2^ or ≥30 kg/m^2^ with metabolic disease [5], is currently the most effective treatment for the management of obesity and related comorbidities [4,6,7].

Despite the remarkable effects of BS on the treatment of obesity, it should be noted that weight loss-related outcomes after this procedure may be influenced by several preoperative factors, including genetic and neurohormonal factors, which have been postulated to affect postoperative weight loss [8,9]. Recently, we showed that the gut microbiome may have a role in the success of BS in terms of percentage excess weight loss (%EWL) [10]. On the other hand, different studies have evaluated the roles of clinical factors, such as baseline weight, age, or sex, in the success of postoperative weight loss [11,12]. Interestingly, some studies have reported that different preoperative comorbidities associated with obesity, including type 2 diabetes (T2D), may lead to lower weight loss following BS [13,14].

Metabolic dysfunction-associated steatotic liver disease (MASLD) often coexists with obesity and other metabolic comorbidities [15,16]. It is associated with an increased risk for cardiovascular disease [17] and has become the first cause of liver transplantation in Western countries [18]. It has been demonstrated that BS is effective for the treatment of MASLD [19,20,21], although only a few studies have evaluated the role of MASLD as a potential predictor of weight loss after BS. In this regard, a previous study conducted in 143 participants with obesity (all of them undergoing gastric bypass) showed that the presence of MASLD before surgery was associated with lower weight loss in the short term following the intervention [22]. However, studies assessing the influence of biopsy-proven MASLD on weight loss after sleeve gastrectomy (SG), the most commonly performed bariatric procedure worldwide, are lacking.

Therefore, in this study, we evaluate the role of MASLD and the different stages of the disease, including metabolic dysfunction-associated steatohepatitis (MASH), in predicting weight loss after SG in patients with obesity.

## 2. Materials and Methods

### 2.1. Study Design and Participants

This was a prospective observational study that included 163 consecutive participants with obesity undergoing SG at Virgen de la Victoria University Hospital from April 2018 to December 2022, with an available intraoperative liver biopsy. All participants underwent laparoscopic sleeve gastrectomy according to international indications [23] and followed a standardized Enhanced Recovery After Surgery (ERAS) protocol for postoperative care [24].

Eligibility criteria to participate in this study included an age of 18–65 years, a BMI ≥ 35 kg/m^2^ or ≥30 kg/m^2^ with relevant comorbidities associated with obesity, laparoscopic SG as the BS technique, and written informed consent to obtain intraoperative liver biopsy. Exclusion criteria were alcohol consumption (>30 g/day in men and >20 g/day in women), use of drugs that could cause liver steatosis, and liver disease different from MASLD.

Participants were categorized as no MASLD, metabolic dysfunction-associated steatotic liver (MASL), or MASH according to the histological evaluation of intraoperative liver biopsies, and were followed up for 1 year after SG.

### 2.2. Histological Evaluation

The histological evaluation of wedge liver biopsies was done by expert liver pathologists. This evaluation was based on the Brunt semi-quantitative classification, including the assessment of liver steatosis, necroinflammatory activity, and fibrosis [25]. Therefore, participants were categorized as no MASLD (no steatosis, no necroinflammatory activity, and no fibrosis), MASL (at least grade 1 steatosis with no necroinflammatory activity nor fibrosis), and MASH (at least grade 1 steatosis with necroinflammatory activity ≥ 1, with or without fibrosis). Further details regarding the histological evaluation can be found elsewhere [26].

### 2.3. Clinical, Anthropometric, and Biochemical Evaluation

Baseline sociodemographic and clinical variables were obtained at a clinical interview. Baseline and 1-year anthropometric data were collected, and included weight, height, and BMI (calculated as weight in kilograms divided by the square of height in meters). Percentage total weight loss (%TWL) at 1 year after SG was calculated by the formula (preoperative weight—weight at 1 year)/preoperative weight × 100. %EWL at 1 year after SG was calculated by the formula (preoperative weight—weight at 1 year)/(preoperative weight—ideal weight) × 100. Ideal weight was calculated for a BMI of 25 kg/m^2^.

Baseline blood samples were collected after a 12 h fast. Serum biochemical parameters were measured by standardized methods (Advia Chemistry XPT autoanalyzer, Siemens Healthcare Diagnostics). Low-density lipoprotein cholesterol (LDL-c) was estimated by Friedewald’s formula [27]. Serum insulin was measured by immunoassay (ADVIA Centaur autoanalyzer, Siemens Healthcare Diagnostics). The formula fasting insulin (μIU/mL) × fasting glucose (mmol/L)/22.5, was used to calculate the homeostasis model assessment of insulin resistance (HOMA-IR) [28].

### 2.4. Statistical Analysis

IBM SPSS statistical software (Version 29.0, IBM Corporation, Chicago, IL, USA) was used for statistical analyses. The normal distribution of variables was assessed using the Kolmogorov–Smirnov test. Comparisons among groups were made using the ANOVA test (continuous variables with a normal distribution) or the Kruskal–Wallis test (continuous variables without a normal distribution), followed by a Bonferroni test. The Pearson’s Chi-squared test was performed to compare proportions. The Student’s *t* test was used to compare continuous variables with a normal distribution between 2 groups. Univariable general linear models were performed considering %TWL or %EWL as the dependent variable and selecting relevant histopathological variables and clinical/biochemical parameters as the fixed factor or covariate. Linear regression models considered %TWL or %EWL as the dependent variable, and different binary histopathological classifications as the independent variable, together with relevant clinical and biochemical parameters for adjustment. Data are given as mean ± standard deviation (SD), or mean (95% confidence interval), unless otherwise indicated. Statistical significance was set for a *p* value < 0.05.

## 3. Results

### 3.1. Basal Characteristics of the Study Population

Data from 163 participants with obesity (58 without MASLD, 38 with MASL, and 67 with MASH) were analyzed. The mean age was 45.7 ± 8.8 years, and 113 (69.3%) were women. The characteristics of the study population at baseline according to MASLD status are shown in Table 1.

Fasting glucose and glycated hemoglobin (HbA1c) levels were higher in patients with MASL or MASH, compared with patients without MASLD. On the other hand, aspartate aminotransferase (AST), alanine aminotransferase (ALT), triglyceride levels, and homeostatic model assessment of insulin resistance (HOMA-IR) values were higher in patients with MASH, compared with patients without MASLD. Data regarding the histopathological evaluation of liver biopsies (i.e., steatosis, necroinflammatory activity, and fibrosis) according to these groups can also be found in Table 1.

### 3.2. Weight Loss After Sleeve Gastrectomy According to MASLD Stage

Weight loss 1 year after SG was evaluated in the different groups of the study population (Table 2). Notably, although no significant differences among the three groups were found for %TWL (*p* = 0.079), significant differences were detected for this outcome when MASH and no MASH (i.e., participants with MASL and participants without MASLD) groups were compared (27.3 ± 9.9 vs. 30.7 ± 9.0., respectively *p* = 0.025).

On the other hand, we found significant differences among groups regarding %EWL (*p* = 0.004). Therefore, a lower %EWL following SG was observed for patients with MASH, compared with patients without MASLD (57.4 ± 20.1 vs. 69.4 ± 21.8, *p* = 0.006). No differences were found between patients with MASL and patients without MASLD (67.8 ± 23.1 vs. 69.4 ± 21.8, *p* = 1.000). A non-significant difference was detected between participants with MASH and participants with MASL (57.4 ± 20.1 vs. 67.8 ± 23.1, *p* = 0.052). %EWL and %TWL stratified by sex are shown in Supplementary Table S1.

### 3.3. Histopathological Factors Associated with Weight Loss After Sleeve Gastrectomy

We performed univariable general linear models to explore baseline histopathological factors associated with weight loss following SG (Table 3). First, we found an inverse relationship between the presence of MASH and %TWL after SG [−3.4% (−6.3 to −0.4)] (Table 3A).

Interestingly, the presence of MASLD and MASH were inversely related to %EWL after SG [−8.3% (−15.3 to −1.3), and −11.4% (−18.1 to −4.7), respectively] (Table 3B). Also, the presence of liver fibrosis, or advanced liver fibrosis (≥F2), was inversely associated with %EWL after SG [−10.4% (−17.6 to −3.2), and −13.5% (−23.5 to −3.5), respectively] (Table 3B).

The role of additional baseline clinical and biochemical variables of interest regarding this outcome are also shown in Table 3. We found that age, the presence of T2D or hypertension, and baseline HbA1c were inversely associated with %TWL, whereas a direct association was observed between baseline weight and %TWL (Table 3A). On the other hand, age, baseline weight, BMI, the presence of T2D, and baseline HbA1c were inversely associated with %EWL after SG (Table 3B).

### 3.4. MASH Is Independently Associated with 1-Year Excess Weight Loss but Not with Percentage Total Weight Loss After Sleeve Gastrectomy

Linear regression models considering %EWL after SG as the dependent variable, and the different histopathological parameters, together with other clinically relevant baseline parameters as independent variables, were performed. Notably, the model that better explained %EWL included age, sex, preoperative MASH, preoperative BMI, and HbA1c. Thus, we observed that the presence of MASH was independently associated with a lower %EWL, after adjusting for age, sex, baseline BMI, and baseline HbA1c [−7.1% (−13.6 to −0.5); *p* = 0.035] (Table 4A), with an adjusted R^2^ of 0.21 for the model. However, the association between MASLD, liver fibrosis, or advanced liver fibrosis, and %EWL after SG did not remain significant after adjusting for these variables [−3.1% (−9.9 to 3.7), *p* = 0.364; −6.4% (−13.3 to 0.4), *p* = 0.066; and −8.9% (−18.3 to 0.5), *p* = 0.062, respectively] (Supplementary Table S2).

On the other hand, in the linear regression model considering %TWL as the dependent variable, the association between MASH and %TWL did not remain significant after adjusting for age, sex, baseline weight, and baseline HbA1c [−2.1% (−5.7 to 0.2); *p* = 0.069] (Table 4B).

## 4. Discussion

The main findings of this study suggest that MASLD status may play a role in postoperative %EWL after SG in the short term (1 year). Specifically, we showed that baseline MASH was independently associated with a lower %EWL after SG in our study population. Conversely, the observed inverse association between MASH and %TWL did not remain significant after adjusting for potential confounders. Therefore, our results add relevant information regarding the role of MASLD and its histopathological stages in weight loss-related outcomes after SG, the most commonly performed bariatric procedure globally, which had remained poorly explored.

BS is the most effective treatment for the management of obesity and related comorbidities, including MASLD. As weight loss is the mainstay of treatment of MASLD, substantial weight loss achieved after BS leads to the improvement and even to the resolution of the disease [19,20,29,30]. Moreover, several studies have also reported favorable results in advanced stages of the disease, such as MASH [31] or liver fibrosis [19,32]. Therefore, patients with obesity and different stages of MASLD can benefit from BS [21].

However, only a few studies have assessed the impact of baseline MASLD on weight loss after BS, and were mainly performed in patients undergoing gastric bypass. In a prospective study that involved 143 patients with obesity undergoing laparoscopic gastric bypass with concomitant intraoperative liver biopsies, the non-alcoholic fatty liver disease (NAFLD) activity score was reported to be a predictor of %EWL at 6 months [22]. Nevertheless, some sample size disproportions among groups were found in this study, as only 13 participants without MASLD (9%) were included in the cohort. On the other hand, in a retrospective cohort of patients undergoing Roux-en-Y gastric bypass, Abbassi et al. showed that %EWL and change in BMI were similar in subjects with MASH and MASL [33]. Sabench et al. found that baseline MASH had a different influence on weight loss depending on the surgical technique, as worse outcomes were reported for patients with MASH that underwent SG, but not in the Roux-en-Y gastric bypass group [34]. However, only women aged 30–55 years, and not men, were included in this study, a fact that could limit external validity. In the recent study by Abu-Rumaileh et al., including participants who underwent BS, preexisting MASLD was independently associated with a lower %TWL and %EWL after the intervention [35]. Notably, these data were retrospectively reviewed, and the definition of MASLD was mainly based on non-invasive criteria (i.e., ICD-9 and ICD-10 coding in electronic medical records, and evidence of hepatic steatosis on imaging studies), and only 38 participants (5% of the study population) had available liver biopsies [35]. Since the non-invasive assessment of MASLD has important limitations for the diagnosis of the disease (e.g., the low reliability of ultrasound to detect hepatic steatosis when <20%, or in individuals with a BMI > 40 kg/m^2^) [36], some of the participants of this study might have been misclassified. Indeed, only 221 participants (31% of the study population) were identified with a diagnosis of MASLD at baseline, which contrasts with the reported higher estimated prevalence of the disease in people living with obesity [37]. Therefore, this prevalence might be explained by the absence of an ICD-9 /ICD-10 diagnosis or available/accurate imaging study in some patients, which may not be enough to rule out the presence of MASLD. Moreover, as MASH diagnosis can only be established by liver biopsy, this stage of the disease was not considered in the study.

Regarding non-surgical weight loss, steatohepatitis was a negative predictor of preoperative weight loss in a cohort of patients with obesity undergoing BS [38]. Additional findings also suggest that patients with overweight/obesity and MASLD might be less responsive to non-surgical approaches to the disease, including lifestyle interventions [39], although further research is needed regarding this point. Also, the mechanisms involved in the potential influence of MASLD on post-BS/non-surgical weight loss are yet to be elucidated. In this regard, it could be speculated that some differences in the gut–liver–brain axis between subjects with and without MASLD might play a role [40]. Another possible explanation of our results might be related to insulin resistance or different hormonal responses following BS, including GLP-1 secretion [41]. Indeed, less weight loss after BS has been observed in other metabolic comorbidities, such as T2D, and some of these mechanisms may play a role [13,42]. However, further research is needed.

Despite the fact that our results suggest that a lower %EWL following SG may be expected in patients with obesity and baseline MASH, it should be noted that, although statistically significant, differences between subjects with and without MASH regarding this outcome were relatively small after adjusting for potential confounders. In fact, participants with baseline MASH achieved a mean %EWL > 50% after SG. Also, clinical differences in %TWL between participants with and without MASH were moderate in this study, and the association between preoperative MASH and %TWL did not remain significant after adjusting for confounders. Therefore, our findings reinforce the fact that BS, including SG, is effective in patients with MASLD (including MASH stage) in terms of weight loss.

On the other hand, we also evaluated the role of histopathological liver fibrosis in %EWL after SG. Although liver fibrosis and advanced liver fibrosis were also associated with lower %EWL in crude models, they did not remain significant after adjusting for confounders. However, a trend towards significance was observed for these two histopathological parameters. In this regard, recent results from the Cologne cohort (including patients undergoing gastric bypass, but not SG) showed that baseline histological fibrosis did not predict %TWL [43]. Given that only a limited proportion of participants had liver fibrosis or advanced liver fibrosis in our study, these results should be cautiously interpreted, and larger studies are needed to evaluate the impact of liver fibrosis on weight loss after SG.

Some of the strengths of this study are its prospective design and the criteria for defining MASLD, which was based on liver biopsy, the gold standard technique for diagnosing the disease. However, despite these strengths, several limitations should be acknowledged. First, sex imbalance should be taken into account, as a predominance of women (69.3%) was observed in our cohort, although these proportions are similar to those observed in clinical practice in patients undergoing BS. Also, the evaluation of weight loss after SG was only considered in the short term. The sample size was relatively small after sub-grouping, which reduced statistical power, especially when analyzing liver fibrosis. Therefore, long-term, large-scale, biopsy-based prospective studies are needed to confirm these results. Furthermore, future perspectives in this area may include evaluating the role of MASLD in weight loss maintenance or weight regain after BS in the long term. Finally, for ethical reasons, no postoperative biopsies were obtained. As previous reports have shown that the persistence of MASH after BS might be associated with less weight loss following the intervention, and changes in liver histology may affect weight loss [32], this could be an important point to consider, which was not evaluated in our study and could have impacted our results.

## 5. Conclusions

In a cohort of patients with obesity undergoing SG, baseline MASH was an independent predictor of lower %EWL, but not %TWL, after the intervention. Further research is needed to unravel the potential mechanisms involved in this association.

## Figures and Tables

**Table 1:** Basal characteristics of the study population according to metabolic dysfunction-associated steatotic liver disease (MASLD) stage.

	No MASLD (n = 58)	MASL (n = 38)	MASH (n = 67)	*p* Value
Sex (F, M)	41/17	27/11	45/22	0.882
Age (years)	43.6 ± 9.2 ^a^	45.8 ± 7.7 ^ab^	47.4 ± 8.7 ^b^	0.048
Weight (kg)	128.8 ± 19.0	130.9 ± 19.3	137.3 ± 26.6	0.204
BMI (kg/m^2^)	46.6 ± 5.9	46.9 ± 5.4	48.9 ± 7.1	0.163
Hypertension (n, %)	22 (37.9%)	12 (31.6%)	36 (53.7%)	0.060
SBP (mm Hg)	131.6 ± 21.0	127.1 ± 12.1	131.5 ± 17.0	0.364
DBP (mm Hg)	83.1 ± 12.4	80.6 ± 9.9	82.0 ± 11.2	0.574
Type 2 diabetes (n, %)	16 (27.6%)	13 (34.2%)	32 (47.8%)	0.060
Glucose (mg/dL)	98.8 ± 18.6 ^a^	107.6 ± 20.0 ^b^	109.1 ± 24.5 ^b^	0.006
HbA1c (%)	5.6 ± 0.7 ^a^	5.9 ± 0.7 ^b^	6.2 ± 1.3 ^b^	0.008
Insulin (μIU/mL)	16.5 ± 8.2 ^a^	17.8 ± 10.0 ^ab^	23.0 ± 13.5 ^b^	0.010
HOMA-IR	4.0 ± 2.1 ^a^	4.7 ± 2.6 ^ab^	6.4 ± 4.6 ^b^	0.003
Cholesterol (mg/dL)	182.0 ± 42.5	185.0 ± 37.1	188.4 ± 39.6	0.680
HDL-C (mg/dL)	44.5 ± 12.4	44.4 ± 13.9	42.7 ± 12.1	0.707
LDL-C (mg/dL)	113.1 ± 38.1	114.6 ± 27.0	115.4 ± 32.6	0.929
Triglycerides (mg/dL)	125.4 ± 63.8 ^a^	137.1 ± 77.4 ^ab^	156.9 ± 72.6 ^b^	0.011
AST (U/L)	25.4 ± 16.1 ^a^	28.1 ± 9.9 ^ab^	31.1 ± 13.9 ^b^	0.006
ALT (U/L)	28.3 ± 18.3 ^a^	35.3 ± 17.5 ^ab^	38.8 ± 18.1 ^b^	<0.001
AST/ALT ratio	0.9 ± 0.3	0.9 ± 0.4	0.9 ± 0.3	0.167
Albumin (g/dL)	3.9 ± 0.4	3.8 ± 0.4	3.8 ± 0.4	0.499
Platelets (10^3^/μL)	278.9 ± 91.0	253.3 ± 60.1	257.9 ± 75.5	0.626
Histopathological parameters				
Steatosis (grade 0/1/2/3)	58/0/0/0	0/29/2/7	0/39/17/11	<0.001
Necroinflammatory activity				
(grade 0/1/2/3)	58/0/0/0	38/0/0/0	0/47/19/1	<0.001
Fibrosis (grade 0/1/2/3/4)	58/0/0/0/0	38/0/0/0/0	16/30/12/8/1	<0.001

Data are given as mean ± standard deviation (SD) or n (proportion). Comparisons among groups were performed using an ANOVA test for continuous variables with a normal distribution (i.e., age, DBP, cholesterol, LDL-C, and albumin), or a Kruskal–Wallis test for continuous variables without a normal distribution (i.e., body weight, BMI, SBP, glucose, HbA1c, insulin, HOMA-IR, HDL-C, triglycerides, AST, ALT, AST/ALT ratio, and platelets), followed by a Bonferroni post hoc analysis. To compare proportions, a Pearson’s Chi-squared test was used. Statistical significance was set for a *p* value < 0.05. Different superscript letters denote statistically significant differences within each row between the groups. MASLD, metabolic dysfunction-associated steatotic liver disease; MASL, metabolic dysfunction-associated steatotic liver; MASH, metabolic dysfunction-associated steatohepatitis; F, female; M, male; BMI, body mass index; SBP, systolic blood pressure; DBP, diastolic blood pressure; HbA1c, glycated hemoglobin; HOMA-IR, homeostatic model assessment of insulin resistance; HDL-C, high-density lipoprotein cholesterol; LDL-C, low-density lipoprotein cholesterol; TG, triglycerides; AST, aspartate aminotransferase; ALT, alanine aminotransferase.

**Table 2:** Weight loss outcomes 1 year after sleeve gastrectomy according to metabolic dysfunction-associated steatotic liver disease (MASLD) stage.

	No MASLD (n = 58)	MASL (n = 38)	MASH (n = 67)	*p* Value
Weight (kg)	88.9 ± 17.2 ^a^	91.2 ± 18.5 ^ab^	98.7 ± 18.4 ^b^	0.008
BMI (kg/m^2^)	32.2 ± 5.9 ^a^	32.7 ± 6.6 ^ab^	35.3 ± 5.9 ^b^	0.007
%EWL	69.4 ± 21.8 ^a^	67.8 ± 23.1 ^ab^	57.4 ± 20.1 ^b^	0.004
%TWL	30.9 ± 8.8	30.3 ± 9.3	27.3 ± 9.9	0.079

Data are given as mean ± standard deviation (SD). Comparisons among groups were performed using an ANOVA test for continuous variables with a normal distribution (i.e., weight, %EWL, and %TWL), or a Kruskal–Wallis test for continuous parameters without a normal distribution (i.e., BMI), followed by a Bonferroni post hoc analysis. Statistical significance was set for a *p* value < 0.05. Different superscript letters denote significant differences within each row between the groups. BMI, body mass index; %EWL, percentage excess weight loss; %TWL, percentage total weight loss. %EWL at 1 year after SG was calculated by the formula (preoperative weight—weight at 1 year)/(preoperative weight—ideal weight) × 100. Ideal weight was calculated for a BMI of 25 kg/m^2^. %TWL at 1 year after SG was calculated by the formula (preoperative weight—weight at 1 year)/preoperative weight × 100.

**Table 3:** (**A**) Univariable general linear model (unadjusted) for predicting percentage total weight loss (%TWL) 1 year after sleeve gastrectomy according to histopathological classifications and clinical/biochemical parameters. (**B**) Univariable general linear model (unadjusted) for predicting percentage excess weight loss (%EWL) 1 year after sleeve gastrectomy according to histopathological classifications and clinical/biochemical parameters.

(A)			
	Beta	95% CI	*p* Value
MASLD (yes vs. no)	−2.5	(−5.6, 0.5)	0.105
MASH (yes vs. no)	−3.4	(−6.3, −0.4)	0.025
Liver fibrosis (yes vs. no)	−2.4	(−5.6, 0.7)	0.129
Liver fibrosis ≥ F2 (yes vs. no)	−4.3	(−8.7, 0.1)	0.053
Age (years)	−0.39	(−0.55, −0.23)	<0.001
Sex (female vs. male)	−0.57	(−3.77, 2.64)	0.728
Baseline weight (kg)	0.07	(0.01, 0.14)	0.029
Baseline BMI (kg/m^2^)	0.16	(−0.06, 0.39)	0.152
Baseline diabetes (yes vs. no)	−4.5	(−7.5, −1.6)	0.003
Baseline HbA1c (%)	−1.5	(−2.9, −0.1)	0.039
Baseline hypertension (yes vs. no)	−3.0	(−6.0, −0.1)	0.046
Baseline triglycerides (mg/dL)	−0.02	(−0.01, 0.04)	0.073
Baseline AST (U/L)	−0.03	(−0.13, 0.08)	0.598
Baseline ALT (U/L)	0.03	(−0.05, 0.11)	0.460
**(B)**			
	**Beta**	**95% CI**	***p* Value**
MASLD (yes vs. no)	−8.3	(−15.3, −1.3)	0.021
MASH (yes vs. no)	−11.4	(−18.1, −4.7)	0.001
Liver fibrosis (yes vs. no)	−10.4	(−17.6, −3.2)	0.005
Liver fibrosis ≥ F2 (yes vs. no)	−13.5	(−23.5, −3.5)	0.008
Age (years)	−0.64	(−1.00, −0.26)	0.001
Sex (female vs. male)	−0.99	(−8.40, 6.42)	0.793
Baseline weight (kg)	−0.17	(−0.31, −0.02)	0.030
Baseline BMI (kg/m^2^)	−1.07	(−1.57, −0.58)	<0.001
Baseline diabetes (yes vs. no)	−8.9	(−15.9, −2.0)	0.012
Baseline HbA1c (%)	−4.4	(−7.7, −1.2)	0.008
Baseline hypertension (yes vs. no)	−4.0	(−10.9, 2.9)	0.253
Baseline triglycerides (mg/dL)	−0.03	(−0.08, 0.02)	0.206
Baseline AST (U/L)	−0.12	(−0.37, 0.13)	0.341
Baseline ALT (U/L)	−0.01	(−0.20, 0.18)	0.937

CI, confidence interval; MASLD, metabolic dysfunction-associated steatotic liver disease; MASH, metabolic dysfunction-associated steatohepatitis; BMI, body mass index; HbA1c, glycated hemoglobin; AST, aspartate aminotransferase; ALT, alanine aminotransferase. Beta values denote the coefficient of the general linear model.

**Table 4:** (**A**) Linear regression model for predicting percentage excess weight loss (%EWL) 1 year after sleeve gastrectomy (dependent variable) according to MASH status (adjusted for age, sex, baseline BMI, and baseline HbA1c). (**B**) Linear regression model for predicting percentage total weight loss (%TWL) 1 year after sleeve gastrectomy (dependent variable) according to MASH status (adjusted for age, sex, baseline weight, and baseline HbA1c).

(A)			
	Beta	95% CI	*p* Value
MASH (yes vs. no)	−7.1	(−13.6, −0.5)	0.035
Age (years)	−0.7	(−1.1, −0.3)	<0.001
Sex (female vs. male)	−1.2	(−7.9, 5.5)	0.729
Baseline BMI (kg/m^2^)	−1.2	(−1.7, −0.7)	<0.001
Baseline HbA1c (%)	−0.8	(−4.0, 2.4)	0.602
**(B)**			
	**Beta**	**95% CI**	***p* Value**
MASH (yes vs. no)	−2.7	(−5.7, 0.2)	0.069
Age (years)	−0.3	(−0.5, −0.2)	<0.001
Sex (female vs. male)	0.4	(−2.9, 3.8)	0.796
Baseline weight (kg)	0.06	(−0.01, 0.13)	0.115
Baseline HbA1c (%)	−0.6	(−2.0, 0.8)	0.386

MASH, metabolic dysfunction-associated steatohepatitis; BMI, body mass index; HbA1c, glycated hemoglobin. Beta values denote the coefficient of the linear regression model.

## Data Availability

Data will be available on reasonable request to the corresponding authors due to ethical reasons.

## Supplementary Materials

The following supporting information can be downloaded at https://www.mdpi.com/article/10.3390/nu16223857/s1; Table S1: Weight loss outcomes 1 year after sleeve gastrectomy according to metabolic dysfunction-associated steatotic liver disease (MASLD) stage (stratified by sex); Table S2: Beta, 95% confidence interval, and *p* value for predicting percentage excess weight loss 1 year after sleeve gastrectomy according to presence of MASLD/liver fibrosis/liver fibrosis ≥F2, after adjusting for age, sex, baseline BMI, and baseline HbA1c.
