# Supplementary material for: Weight Loss After Sleeve Gastrectomy According to Metabolic Dysfunction-Associated Steatotic Liver Disease Stage in Patients with Obesity: A Liver Biopsy-Based Prospective Study
Source: Nutrients. 2024 Nov 12;16(22):3857. doi: 10.3390/nu16223857 (PMC11597773; doi:10.3390/nu16223857)
Supplement: Supplementary file 1 [file nutrients-16-03857-s001.zip › Supplementary Table S1.pdf]

Supplementary Table S1. Weight loss outcomes 1 year after sleeve gastrectomy according to metabolic dysfunction-associated steatotic liver disease (MASLD) stage (stratified by sex).

	No MASLD (n=41-women, 17-men)	MASL (n= 27-women, 11-men)	MASH (n= 45-women 22-men)	p value
%EWL (women, n= 113)	68.9 ± 21.2 [a]	68.8 ± 24.9 [ab]	56.2 ± 21.9 [b]	0.017
%TWL (women, n= 113)	31.2 ± 9.3 [a]	31.0 ± 9.5 [ab]	26.1 ± 9.7 [b]	0.026
%EWL (men, n= 50)	70.7 ± 21.4	65.4 ± 18.9	59.9 ± 16.0	0.203
%TWL (men, n= 50)	30.2 ± 7.9	28.7 ± 9.2	29.7 ± 9.0	0.915

Data are given as mean ± standard deviation (SD). Comparisons among groups were performed using an ANOVA test, followed by a Bonferroni post-hoc analysis. Statistical significance was set for a p value < 0.05. Different superscript letters denote significant differences within each row between the groups. BMI, body mass index; %EWL, percentage excess weight loss; %TWL, percentage total weight loss. %EWL at 1 year after SG was calculated by the formula: (preoperative weight -weight at 1 year)/ (preoperative weight - ideal weight) x 100. Ideal weight was calculated for a BMI of 25 kg/m^2. %TWL at 1 year after SG was calculated by the formula: (preoperative weight - weight at 1 year)/ preoperative weight x 100.
