# Supplementary material for: Weight Loss After Sleeve Gastrectomy According to Metabolic Dysfunction-Associated Steatotic Liver Disease Stage in Patients with Obesity: A Liver Biopsy-Based Prospective Study
Source: Nutrients. 2024 Nov 12;16(22):3857. doi: 10.3390/nu16223857 (PMC11597773; doi:10.3390/nu16223857)
Supplement: Supplementary file 1 [file nutrients-16-03857-s001.zip › Supplementary Table S2.pdf]

Supplementary Table S2. Beta, 95% confidence interval, and p value for predicting percentage excess weight loss (%EWL) 1 year after sleeve gastrectomy according to the presence of MASLD/liver fibrosis/liver fibrosis ≥ F2, after adjusting for age, sex, baseline BMI, and baseline HbA1c

	Beta	95% CI	p value
MASLD (yes *vs.* no)	-3.1	(-9.9, 3.7)	0.364
Liver fibrosis (yes *vs.* no)	-6.4	(-13.3, 0.4)	0.066
Liver fibrosis ≥ F2 (yes *vs.* no)	-8.9	(-18.3, 0.5)	0.062

CI, confidence interval, MASLD, metabolic dysfunction-associated steatotic liver disease; ≥ F2, liver fibrosis stage 2 or higher; BMI, body mass index; HbA1c, glycated hemoglobin. Given values are adjusted for age, sex, baseline BMI, and baseline HbA1c in the linear regression analysis. Beta values denote the coefficient of the linear regression model.
